# A CRE1- regulated cluster is responsible for light dependent production of dihydrotrichotetronin in *Trichoderma reesei*

**DOI:** 10.1371/journal.pone.0182530

**Published:** 2017-08-15

**Authors:** Alberto Alonso Monroy, Eva Stappler, Andre Schuster, Michael Sulyok, Monika Schmoll

**Affiliations:** 1 AIT - Austrian Institute of Technology GmbH, Center for Health & Bioresources, Tulln, Austria; 2 TU Wien, Institute of Chemical Engineering, Research Area Molecular Biotechnology, Vienna, Austria; 3 University of Natural Resources and Life Sciences Vienna, Department for Agrobiotechnology (IFA-Tulln), Center for Analytical Chemistry, Tulln, Austria; Ruhr-Universitat Bochum, GERMANY

## Abstract

Changing light conditions, caused by the rotation of earth resulting in day and night or growth on the surface or within a substrate, result in considerably altered physiological processes in fungi. For the biotechnological workhorse *Trichoderma reesei*, regulation of glycoside hydrolase gene expression, especially cellulase expression was shown to be a target of light dependent gene regulation. Analysis of regulatory targets of the carbon catabolite repressor CRE1 under cellulase inducing conditions revealed a secondary metabolite cluster to be differentially regulated in light and darkness and by photoreceptors. We found that this cluster is involved in production of trichodimerol and that the two polyketide synthases of the cluster are essential for biosynthesis of dihydrotrichotetronine (syn. bislongiquinolide or bisorbibutenolide). Additionally, an indirect influence on production of the peptaibol antibiotic paracelsin was observed. The two polyketide synthetase genes as well as the monooxygenase gene of the cluster were found to be connected at the level of transcription in a positive feedback cycle in darkness, but negative feedback in light, indicating a cellular sensing and response mechanism for the products of these enzymes. The transcription factor TR_102497/YPR2 residing within the cluster regulates the cluster genes in a light dependent manner. Additionally, an interrelationship of this cluster with regulation of cellulase gene expression was detected. Hence the regulatory connection between primary and secondary metabolism appears more widespread than previously assumed, indicating a sophisticated distribution of resources either to degradation of substrate (feed) or to antagonism of competitors (fight), which is influenced by light.

## Introduction

In their natural habitat, fungi constantly face the challenge to outcompete other organisms in complex ecosystems. Therefore they developed powerful enzyme systems for degradation of substrates, which provide for fast growth and efficient colonization of their environment [1]. However, fungi also evolved the ability to kill, or at least inhibit the growth of their competitors by producing a versatile array of secondary metabolites [2]. Application of these different survival utilities has to be tightly controlled in order to balance the assignment of resources for feeding to succeed by superior growth or fighting to decrease the chances of survival for competitors.

Due to its high capacity for cellulose degradation, which is also industrially exploited [3], the filamentous fungus *Trichoderma reesei* (anamorph of *Hypocrea jecorina*) has become a model organism for plant cell wall degradation [4, 5]. Traditionally, this species was also used as a model for studying light responses [6]. Cellulases [7] as well as numerous glycoside hydrolases are regulated in dependence of the light status in *T*. *reesei* [8–10] and the photoreceptors BLR1, BLR2 and ENV1. Also the photoreceptor homologues in *Neurospora crassa*, WC-1, WC-2 and VVD were shown to regulate cellulase gene expression in [11]. *T*. *reesei* BLR1 and BLR2 receive blue light as a signal and exert their function as GATA-type transcription factors [6, 12]. They were shown to have functions in regulation of plant cell wall degrading enzymes as well as CAZyme genes in general in dependence of light [10, 13] and to influence regulation of the pheromone system in *T*. *reesei* [14]. ENV1 is a PAS domain protein acting as a photoreceptor and is regulated by BLR1 and BLR2 [12]. Also ENV1 impacts CAZyme gene expression [10], pheromone response [14] and sexual development [14, 15] and is assumed to exert its function in part via the cAMP pathway [16, 17]. ENV1 further connects light response to oxidative stress response due to an evolutionary conserved amino acid alteration compared to *N*. *crassa* [18].

However, also numerous components of the signaling pathways are known to impact cellulase regulation [19, 20] indicating that many extracellular signals causing this regulation still remain to be discovered.

Production of extracellular enzymes is an energy-consuming process and only initiated when needed [21]. In the presence of plant cell wall components, expression of hydrolytic enzymes is induced, which act synergistically for degradation of the complex polymers present in this substrate. As these polymers are too large to enter the fungal cell, small products of their hydrolysis act as signaling molecules for the presence of degradable plant material [22, 23]. One of the crucial mechanisms for regulation of enzyme production needed for substrate utilization is carbon catabolite repression (CCR). CCR prevents biosynthesis of numerous hydrolytic enzymes involved in degradation of complex polysaccharides, if an easily metabolizable carbon source is available [24, 25]. Thereby, the *T*. *reesei* carbon catabolite repressor CRE1 can act positively or negatively on gene regulation and the extent of this effect is also dependent on the growth rate in many cases [26].

CRE-1 was shown to be a direct target of the white collar complex (WCC) in *N*. *crassa* upon growth on sucrose [27] and its transcript rapidly increases upon onset of illumination [28]. Furthermore, *cre-1* is also regulated by the WCC *N*. *crassa* on cellulose [11] and its deletion in *N*. *crassa* leads to considerably increased cellulase activity on cellulose [11, 29].

Usually, secondary metabolites are preferentially produced after the active growth phase, if nutrients in the environment become limiting or if environmental conditions such as humidity, temperature, UV irradiation or pH threaten the functionality of the fungal cell [30]. In fungi, biosynthesis of secondary metabolites is organized via regulation of specific gene clusters [31]. However, many of these gene clusters, which became obvious during analysis of fungal genomes, appear to be silent under common laboratory conditions. Therefore, recent research efforts concentrate on elucidation of regulation of secondary metabolite clusters and investigation of environmental signals initiating activation of such silent clusters and “cryptic” pathways connected to them [32, 33]. In this respect, especially overexpression of a putative regulator of a predicted but silent cluster proved effective [34]. These efforts also indicate a regulatory crosstalk between different secondary metabolite pathways, due to activation of more than one cluster upon overexpression of a regulator (for example LaeA) [35].

As other fungi, species of the genus *Trichoderma* apply chemical warfare to defend their territory [36]. More than 100 such metabolites have been described for *Trichoderma* spp. and range from potential antibiotics to mycotoxins as well as volatile organic compounds [37, 38]. For the biotechnological workhorse *Trichoderma reesei*, the trichothecene toxin trichodermin [39] as well as the peptaibol antibiotic paracelsin [40] have been described. Trichodermin is much less toxic than most other metabolites of the group trichothecene toxins [39]. However, only limited data is available on regulation of individual secondary metabolites in *T*. *reesei* (see also below).

Two regulators important for production of the yellow pigment produced by *T*. *reesei*, YPR1 and YPR2, were identified. A metabolite produced by the gene cluster located next to the genes encoding YPR1 and YPR2 was determined to be sorbicillin [41].

The genome of *T*. *reesei* contains 11 polyketide synthases [42] and several non-ribosomal peptide synthetases [43]. Although this number is small compared to other fungi [42], their presence indicates a considerable potential for production of secondary metabolites. Interestingly, analysis of the genome also revealed that plant cell wall degrading enzymes of *T*. *reesei* are often found in clusters along with genes involved in secondary metabolism [43]. Consequently, it is reasonable to assume that *T*. *reesei* evolved a mechanism for balancing the operation of primary and secondary metabolism during its life cycle. Indeed, the transcription factor XPP1, for which previously a function in xylanase regulation was reported [44], was suggested to act as a switch between primary and secondary metabolism [45]. Thereby, lack of XPP1 causes both increased diversity and quantity in produced secondary metabolites in T. reesei upon growth on glucose [45]. Moreover, XPP1 regulates transcript levels of different polyketide synthase encoding genes including TR_73618 and TR_73621, which are located close to YPR1 and YPR2 in the genome of *T*. *reesei* [45].

It was shown that production of secondary metabolites as well as the light dependence of this process is strongly dependent on the carbon source in fungi. Even the concentration of the carbon source in the cultivation medium can switch the preference for secondary metabolite production from light to darkness in *Aspergillus nidulans*. This process is regulated by VeA and its associated proteins including the photoreceptors LreA and LreB as well as the phytochrome FphA ([46] and references therein).

Here we investigated the connection between cellulase regulation and secondary metabolite production with respect to a light- and photoreceptor regulated gene cluster. We show a connection between secondary metabolite production and cellulase regulation as well as a light dependent feedback regulation of the biosynthetic genes within the cluster. Our findings further revealed that the genes of this cluster are required for production of dihydrotrichotetronin.

## Results

### Regulatory targets of CRE1 are different in light and darkness

**Fig 1:**
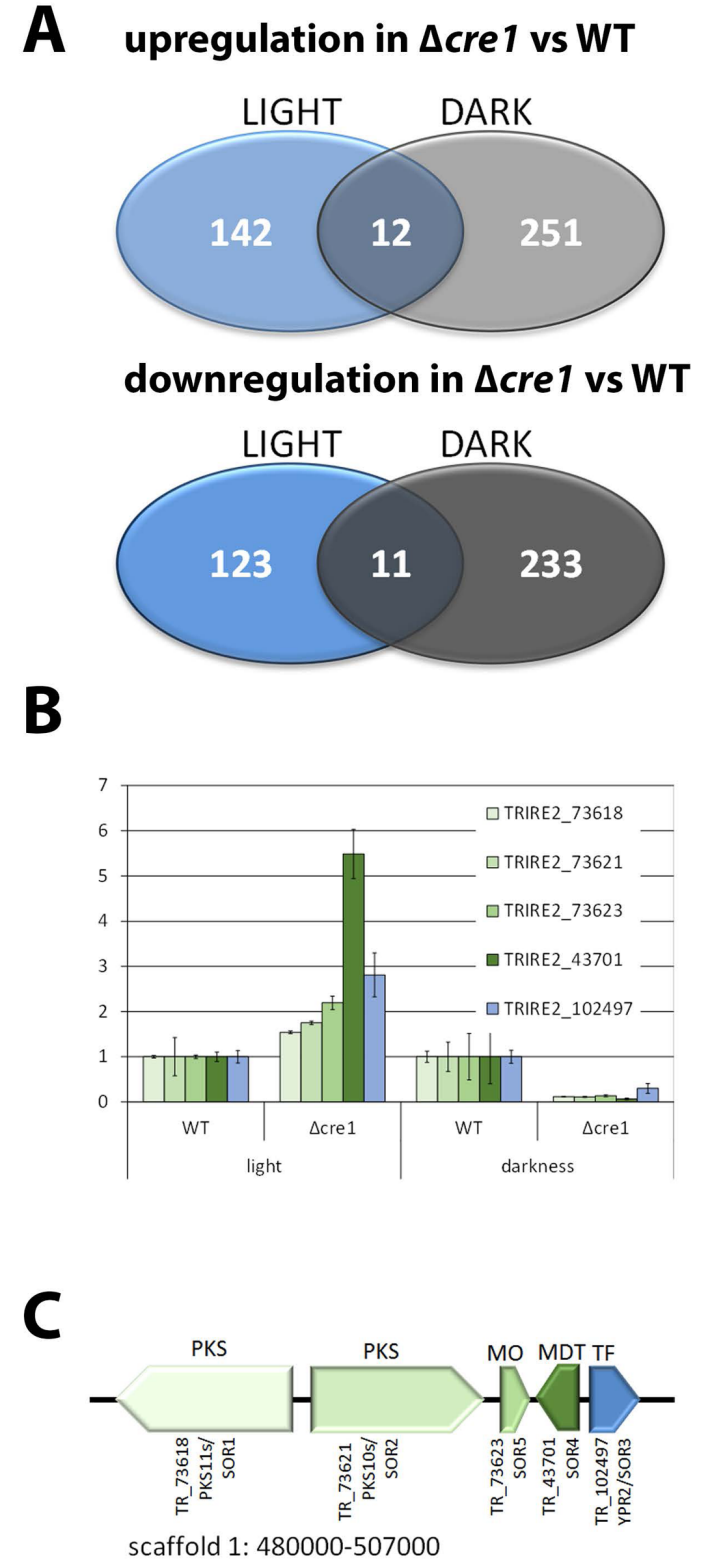
Light dependent regulation by CRE1 and the CRE1-regulated cluster. Transcriptome analysis of Δ*cre1* was done in comparison to QM9414 as wild-type in constant light and constant darkness upon growth on microcrystalline cellulose as carbon source for 72 hours. (A) Venn diagrams showing positive and negative gene regulation in a strain lacking *cre1* in light and darkness upon growth on cellulose. (B) Regulation of cluster genes by CRE1 in light and darkness related to the respective wildtype strain under the same conditions. (C) Schematic representation of the cluster genes along with protein IDs as assigned in JGI (http://genome.jgi.doe.gov/Trire2/Trire2.home.html) along with protein designations assigned previously [5, 41, 48].

We investigated the the effect of a loss of CRE1 on light/dark regulated gene expression upon growth on cellulose. Therefore we cultivated Δ*cre1* and the wild-type strain in Mandels Andreotti minimal medium with microcrystalline cellulose as carbon source for 72 hours in constant light (1800 lux, white light) or in constant darkness. Transcript levels of 263 genes increased in Δ*cre1* as compared to the wild-type in darkness and 154 increased in Δ*cre1* in comparison to the wild-type in light, while a decrease of transcript levels was observed for 244 genes in darkness and 134 genes in light. Only 12 genes were upregulated in darkness and light and 11 genes showed a decrease in both conditions (Fig 1A; S1 File). Genes upregulated in darkness are enriched in functions of metabolism (p-value 5.42E-04), amino acid metabolism (p-value 1.32E-03), nitrogen, sulfur and selenium metabolism (p-value 2.37E-03), ion transport (p-value 1.99E-03) and transport facilities (p-value 8.30E-05). In light, upregulated genes show an enrichment in metabolism (p-value 2.79E-04) as well and further in nitrogen, sulfur and selenium metabolism (p-value 6.32E-06) in transport facilities (p-value 4.47E-03), cellular import (p-value 1.20E-03). Hence, CRE1 consistently negatively regulates metabolic genes and transport in light and darkness, albeit the targets of these functional shifts are not the same in light and darkness.

Positive effects of CRE1 on gene regulation showed a striking difference in targeted functions in light and darkness. Although again, several metabolic genes were regulated, no significant enrichment was observed within the genes downregulated in the mutant strain in light. In darkness, genes involved in sugar, glucoside, polyol and carboxylate catabolism and anabolism (p-values <3.35E-03) were enriched. However, the most striking difference was found in regulation of genes involved in protein synthesis (45 genes) i. e. ribosomal proteins and genes involved in ribosome biogenesis and translation with p-values below 7E-22, which only occurs in darkness.

Hence our analysis confirms the function of CRE1 in regulation of metabolism also upon growth on cellulose. Additionally, gene regulation by CRE1 is specific for light and darkness with surprisingly few genes being regulated in light as well as darkness (Fig 1A).

### Genes regulated by CRE1 are clustered in the genome

Evaluation of the genomic loci of genes up- or down-regulated by CRE1 in light and darkness revealed a non random distribution of 259 genes, which were assigned to 36 genomic clusters (S2 File). In several cases, these clusters contained CAZyme encoding genes. Interestingly, again a strong enrichment among the genes in these 36 clusters in functions of protein synthesis, ribosome biogenesis and translation (up to p-values of E-09) was found.

**Fig 2:**
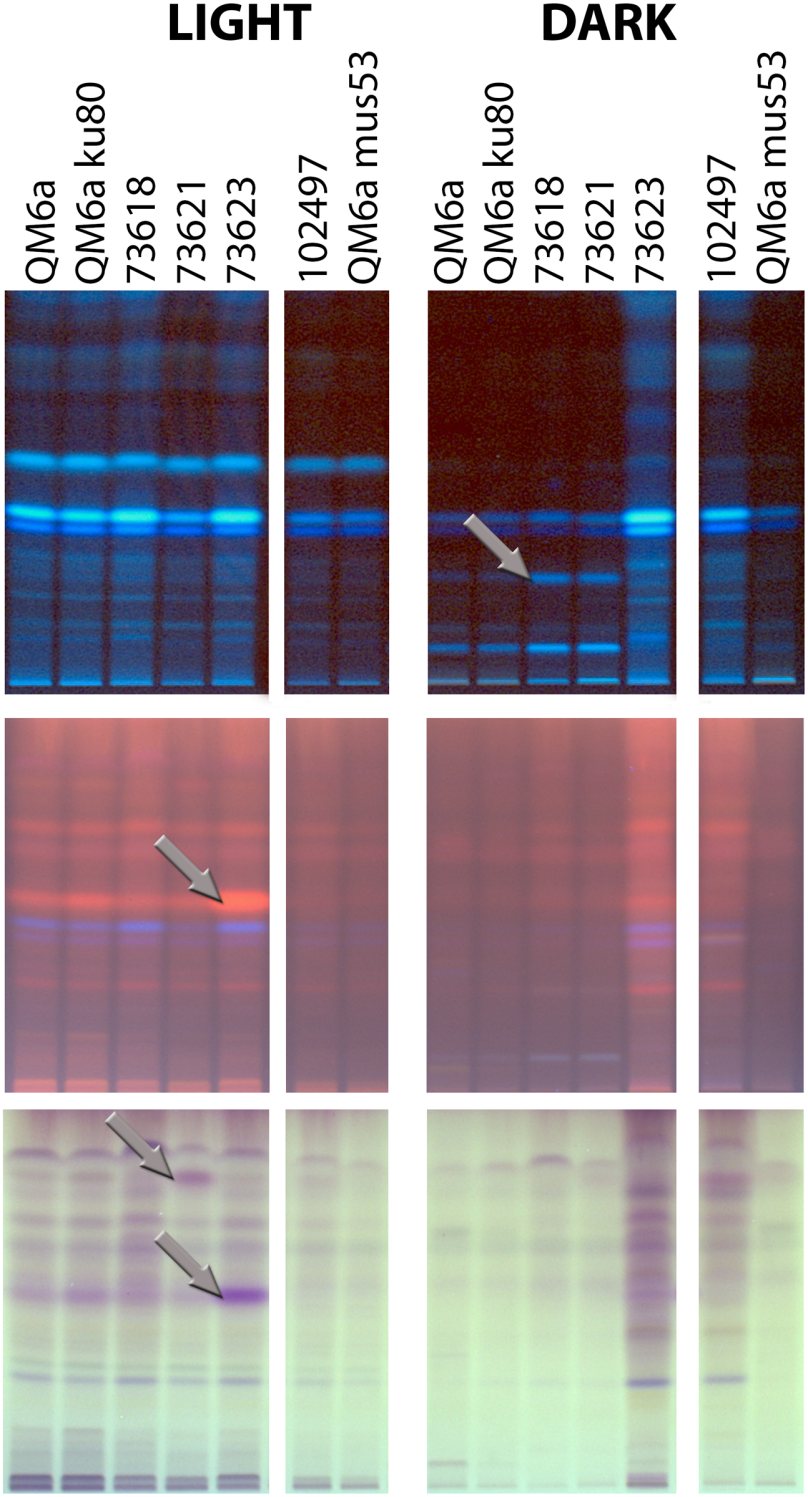
Secondary metabolite patterns in deletion mutants. HPTLC (high performance thin layer chromatography) analysis of secondary metabolites secreted by mutant strains in the LCS cluster upon growth on cellulose in light or darkness. Numbers represent protein IDs of genes deleted in the respective analyzed strain. Samples are adjusted to biomass produced and hence represent secondary metabolites produced by equal amounts of biomass. The three panels represent different methods of visualization of the same metabolite patterns: Upper panel: Remission at 366 nm; middle panel: derivatized, remission at 366 nm, lower panel: derivatized, transmission visible light. The analysis of light- and dark samples was done in parallel on the same HPTLC plate and consequently signal strengths in light and dark are comparable. Arrows highlight bands with altered signal strength compared to wildtype. Three biological replicates were analyzed and a representative sample is shown.

Among these clusters was also one comprising secondary metabolism genes (cluster 1 in S2 File) which resides in a genomic area previously described as a region of increased CAZyme density ([43]; Figure 2a therein). Part of the cluster we found and which is described in [43] was recently described to be responsible for production of a sorbicillin component in *T*. *reesei* [41] and in *Penicillium chrysogenum* [47] and is regulated by XPP1 in *T*. *reesei* [45].

### CRE1 differentially regulates a secondary metabolite cluster in light and darkness

Our previous data showed that this cluster 1 (S2 File) overlaps with a light dependently regulated cluster on cellulose [9]. Accordingly, (direct or indirect) regulation of the cluster by CRE1 was negative in light and positive in darkness (Fig 1B). Hence we refer to this cluster sorbicillin- or “SOR” cluster and we selected the two polyketide synthetase genes TR_73618 and TR_73621, the monooxygenase gene TR_73623, the transporter gene TR_43701 and the transcription factor gene TR_102497 for further analysis. Searching 1000 bp of the upstream sequences of these genes all contain putative CRE1 binding motifs (5’ SYGGRG 3’, -874, -813, -426 and -424, relative to the ATG for TR_73618; -852, -349 and -347 for TR_73621, -950, -921 and -273 for TR_73623, -192 for TR_43701 and -726, -660, and -358 for TR_102497). Fig 1C shows the composition of the cluster in *T*. *reesei* along with the gene designations assigned in previous publications [5, 41, 48]. In order to avoid confusion, we will use the unique JGI protein ID number of the respective genes hereafter.

Since the genes in the SOR cluster are regulated in a light dependent manner, we checked transcriptome data on the photoreceptors BLR1, BLR2 and ENV1 grown under similar conditions (GSE36448; [10]) for regulation of the genes in our cluster. We found that the photoreceptors BLR1 and BLR2 negatively regulate the genes of this cluster, while ENV1 exerts positive regulation (Figure A in S3 File; [10]), hence suggesting that this cluster is important for photoadaptation [49–51]. Evaluation of transcriptome data from growth of the wild-type on different carbon sources revealed that the cluster is upregulated upon growth on cellulose and on glucose, but only very low transcript levels are present on glycerol, lactose and sophorose (GSE81365) [9], which is in agreement with detection of a product of the cluster upon growth on glucose as reported previously [41]. The regulation of the SOR cluster genes by components of the heterotrimeric G-protein pathway as found upon re-analysis of transcriptome data from strains lacking the G-protein beta and gamma subunits or a phosducin (GSE27581) [8] supports a connection to nutrient sensing.

These findings suggest that CRE1 is not only the main carbon catabolite repressor, but also involved in light dependent balancing of secondary metabolism and substrate degradation.

### The SOR cluster genes influence secondary metabolite patterns in a light dependent manner

We deleted the genes of the SOR cluster and investigated their functions in secondary metabolism. Therefore we used high performance thin layer chromatography (HPTLC) to obtain a first overview on secondary metabolite patterns secreted by *T*. *reesei* upon growth on cellulose (Fig 2). This analysis showed that the overall amount of secondary metabolites per biomass is lower in darkness for most strains than it is in the light, since the signal strength of most strains including the wild-type QM6a in the “DARK” panel is weaker (Fig 2). In the light, individual bands for TR_73623 and TR_73621 (arrows in Fig 2, “LIGHT” panel) are increased. However, in darkness, we found elevated signal strengths in several bands for the two PKS-encoding genes TR_73618 and TR_73621 (upper “DARK” panel) as well as a general increase of band intensities for the monooxygenase-encoding TR_73623. Deletion of the transcription factor-encoding TR_102497 also caused an increase in visible bands in darkness (Fig 2).

### TR_73618 and TR_73621 are required for the biosynthesis of dihydrotrichotetronine

**Fig 3:**
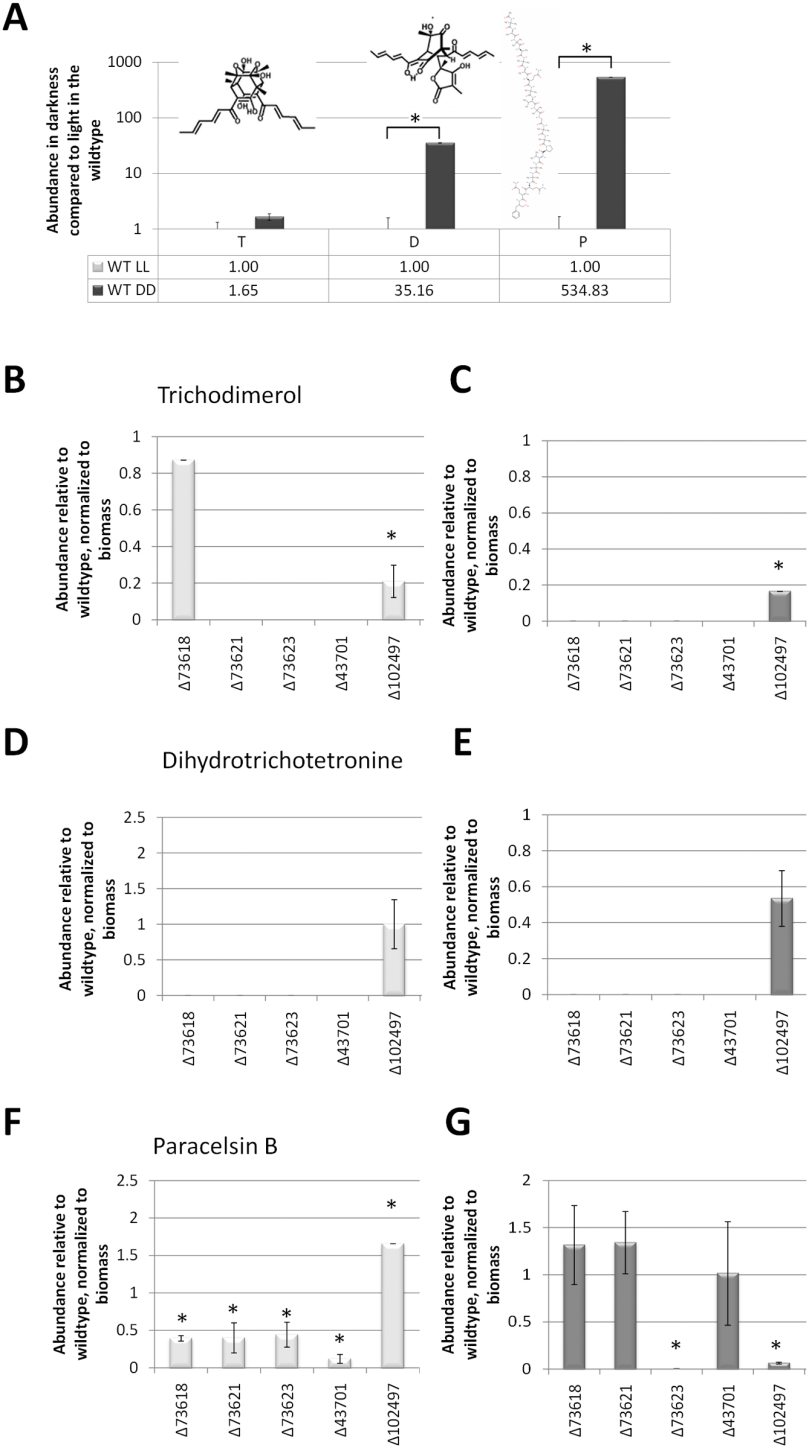
Quantitative mass spectrometry analysis in light and darkness. (A) Quantitative analysis of abundance of trichodimerol (“T”), dihydrotrichotetronin (“D”) and paracelsin B (“P”) in light compared to darkness in wildtype. (B-G) Quantitative analysis of abundance of trichodimerol (B, C), dihydrotrichotetronin (D,E) and paracelsin (F,G) in light (light grey bars) and darkness (dark grey bars) in strains lacking cluster genes upon growth in liquid minimal medium with cellulose as carbon source. Produced metabolites were related to the biomass formed under the respective conditions. Errorbars show standard deviations of two biological replicates. Values with statistically significant difference from wild-type are marked with an asterisk (except for lacking production). Structures show the respective compound.

In order to gain an insight into the nature of the metabolites formed by the SOR cluster, we investigated the secreted metabolites upon growth on cellulose by mass spectrometry using a multimetabolite standard solution for precise metabolite identification and quantification. We found that the amount of trichodimerol only showed an increasing trend in darkness compared to light, while dihydrotrichotetronine and paracelsin levels were strongly increased in darkness (Fig 3A). Deletion of the transcription factor gene *ypr2*/TR_102497 clearly decreased production of trichodimerol in light and darkness, which confirms its function in regulation of the SOR cluster (Fig 3B–3E). Lack of the PKS encoding genes and the monoxygenase and the transporter consistently abolished production of trichodimerol and dihydrotrichotetronin in darkness, while trichodimerol was still detectable in TR_73618 in light (Fig 3B–3E). Hence we conclude that the SOR cluster is required for the production of trichodimerol and dihydrotrichotetronin, with TR_73621 being sufficient for production of trichodimerol, but not dihydrotrichotetronin in the light.

In addition to the function of the cluster in production of these compounds, deletion of SOR cluster genes in part also impacts production of paracelsin in a light dependent manner, but likely in an indirect way. Deletion of *ypr2*/TR_102497 causes somewhat increased paracelsin levels in light, while, all other deletion mutants showed decreased paracelsin levels in light (Fig 3F). In darkness, only TR_73623 and. *ypr2*/TR_102497 were relevant for paracelsin levels and suggest an involvement of TR_73623 in biosynthesis of paracelsin or a precursor as well as direct or indirect regulation of paracelsin biosynthesis by *ypr2*/TR_102497 (Fig 3G).

### YPR2 differentially regulates cluster genes in light and darkness

**Fig 4:**
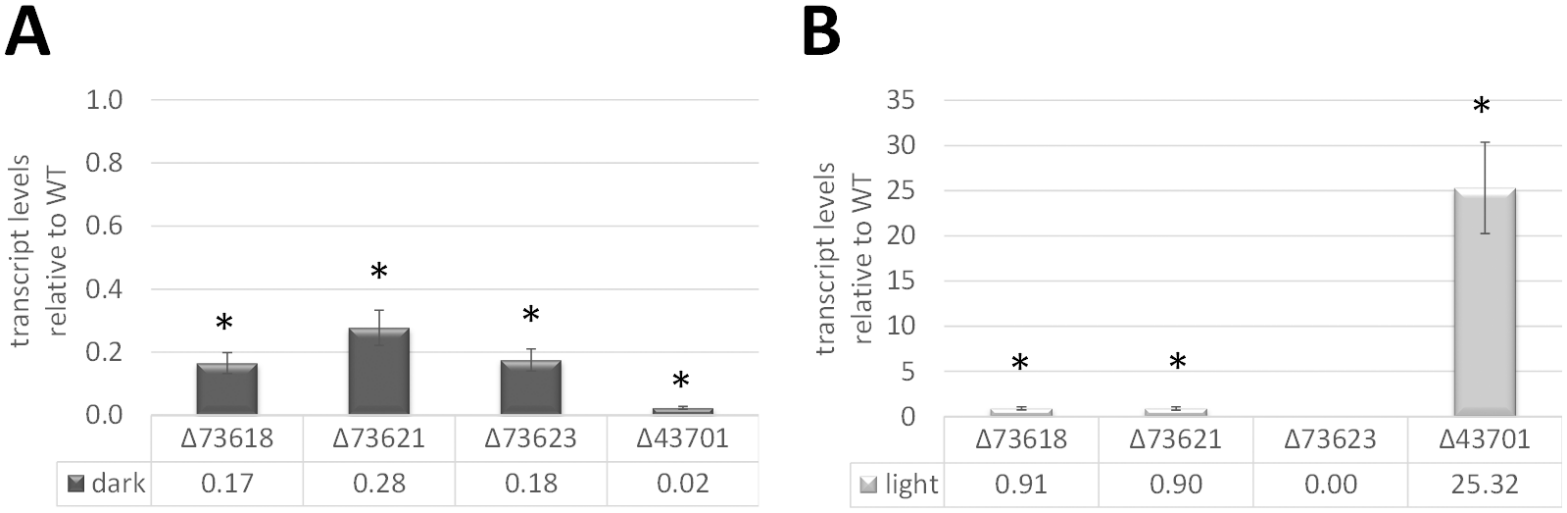
Transcript levels of cluster genes in a strain lacking TR_102497/YPR2. Transcript levels of TR_73618, TR_73621, TR_73623 and TR_43701 were determined by quantitative RT-PCR after growth on cellulose in constant darkness (A) or in constant light (B) for 96 hours and are shown relative to the wildtype. Errorbars show standard deviations of at least three biological replicates and three technical replicates. Values with statistically significant difference from wild-type are marked with an asterisk (except when no transcript was detected at all).

Since HPTLC and mass spectrometry data clearly showed a role of YPR2 in regulation of the secondary metabolites produced by the cluster, we analyzed its role in regulation of the individual genes. Upon growth on cellulose in darkness, we found a strong positive influence of YPR2 on the four other genes in the cluster (Fig 4A). In light, the pks genes were only present at a very low level already in the wild-type and only a minor influence of YPR2 was observed (Fig 4B). For the monooxygenase, however, YPR2 is essential for induction in light and YPR2 has a strongly negative effect on the transporter gene TR_43701 in light (Fig 4B).

### Biosynthetic genes show light dependent mutual regulation

**Fig 5:**
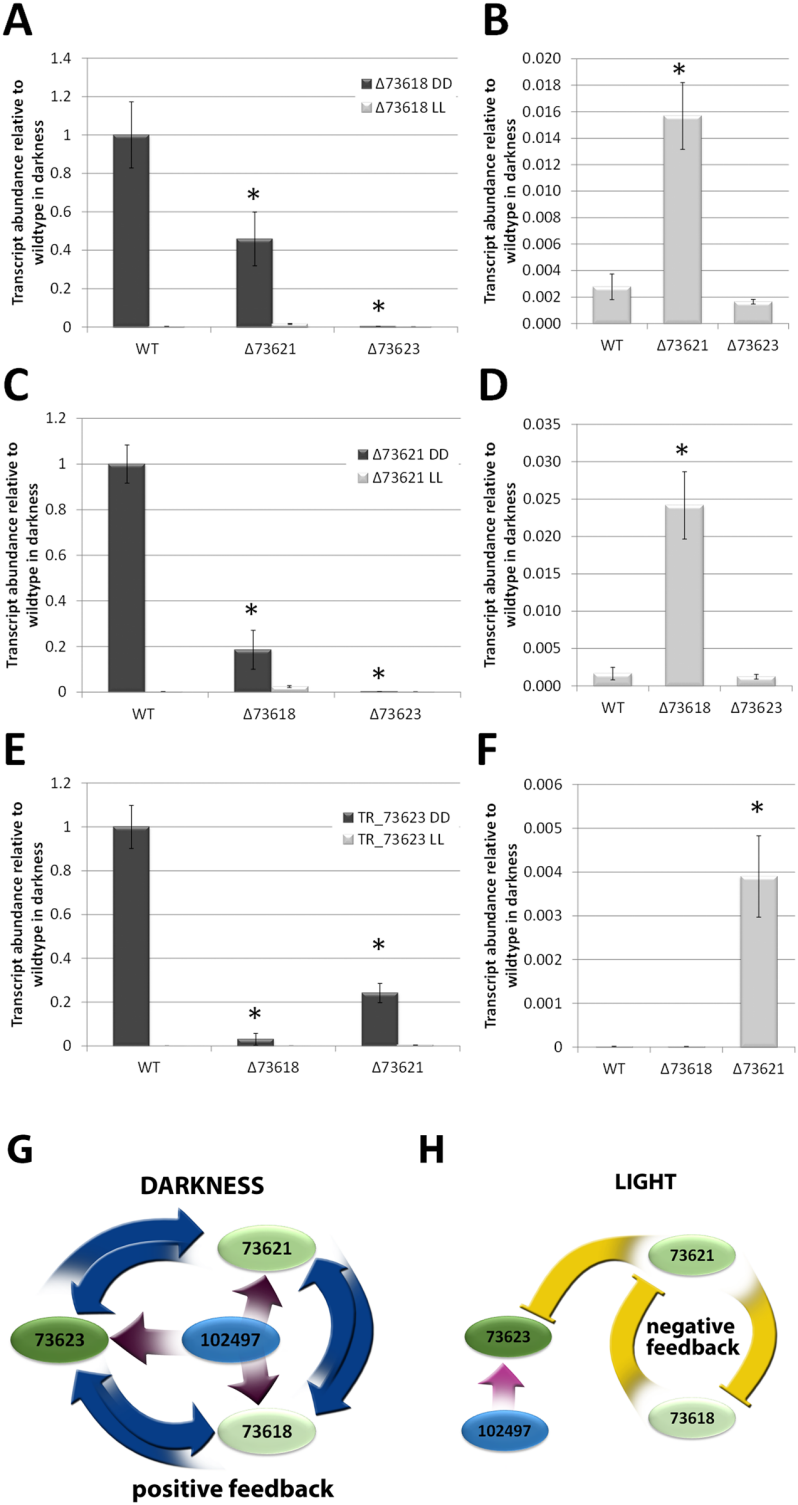
Mutual regulatory response of biosynthetic genes to deletions. The effects of deletions of the biosynthetic genes on transcript levels of TR_73618 (A, B), TR_73621 (C, D) and TR_73623 (E, F) in constant light (LL) or constant darkness (DD) are shown relative to wild-type in darkness. As transcript levels in light are too low to be evaluated next to darkness results (A, C, E), they were also presented separately (B, D, F) with the y-axis showing transcript levels in relation to the wild-type in darkness. Strains were grown in constant light (LL) or constant darkness (DD) on cellulose for 96 hours. Errorbars show standard deviations of at least three biological replicates and three technical replicates. (G, H) Schematic representation of positive and negative feedback of mutual gene regulation in light and darkness. Values with statistically significant difference from wild-type are marked with an asterisk in A,C and E for darkness and in B, D and F for light.

A mutual influence of biosynthetic genes due to a feedback mechanism caused by altered precursor availability seemed possible. In the wild-type we found strongly decreased transcript levels upon growth in light to less than 1% of dark levels for TR_73618, TR_73621 and TR_73623 (Fig 5A–5F), which is in agreement with transcriptome data. Transcript levels of TR_73618 in deletion strains of TR_73621 or TR_73623 were decreased in darkness (Fig 5A) and transcript levels of TR_73621 in darkness strongly decreased in the absence of TR_73618 and were around the extremely low light levels in a strain lacking TR_73623 (Fig 5C and 5D). For TR_73623 decreased transcript abundance was observed in deletion strains of TR_73621 and TR_73618 (Fig 5E). Hence, the function of TR_73623 is required for induction of the pks genes TR_73618 and TR_73621 and the absence of one of the pks genes leads to a decrease in transcript abundance of the other by roughly 5 fold. Consequently, the biosynthetic genes show a mechanism of positive feedback on each other in darkness (Fig 5G).

In light, transcription levels of these three genes were already at a very low level compared to darkness (Fig 5B, 5D and 5F) and the regulatory effects were less dramatic. Deletion of TR_73618 increased transcript levels of TR_73621 and vice versa. For TR_73623 no significant influence on the pks genes was observed, but on the other hand, TR_73621 did have a positive effect on transcript levels of TR_73623 (Fig 5H).

In summary this analysis revealed a positive feedback cycle in darkness and a negative feedback cycle, albeit predominantly comprising the pks genes, in light (Fig 5G and 5H).

### Genes of the SOR cluster influence cellulase regulation

**Fig 6:**
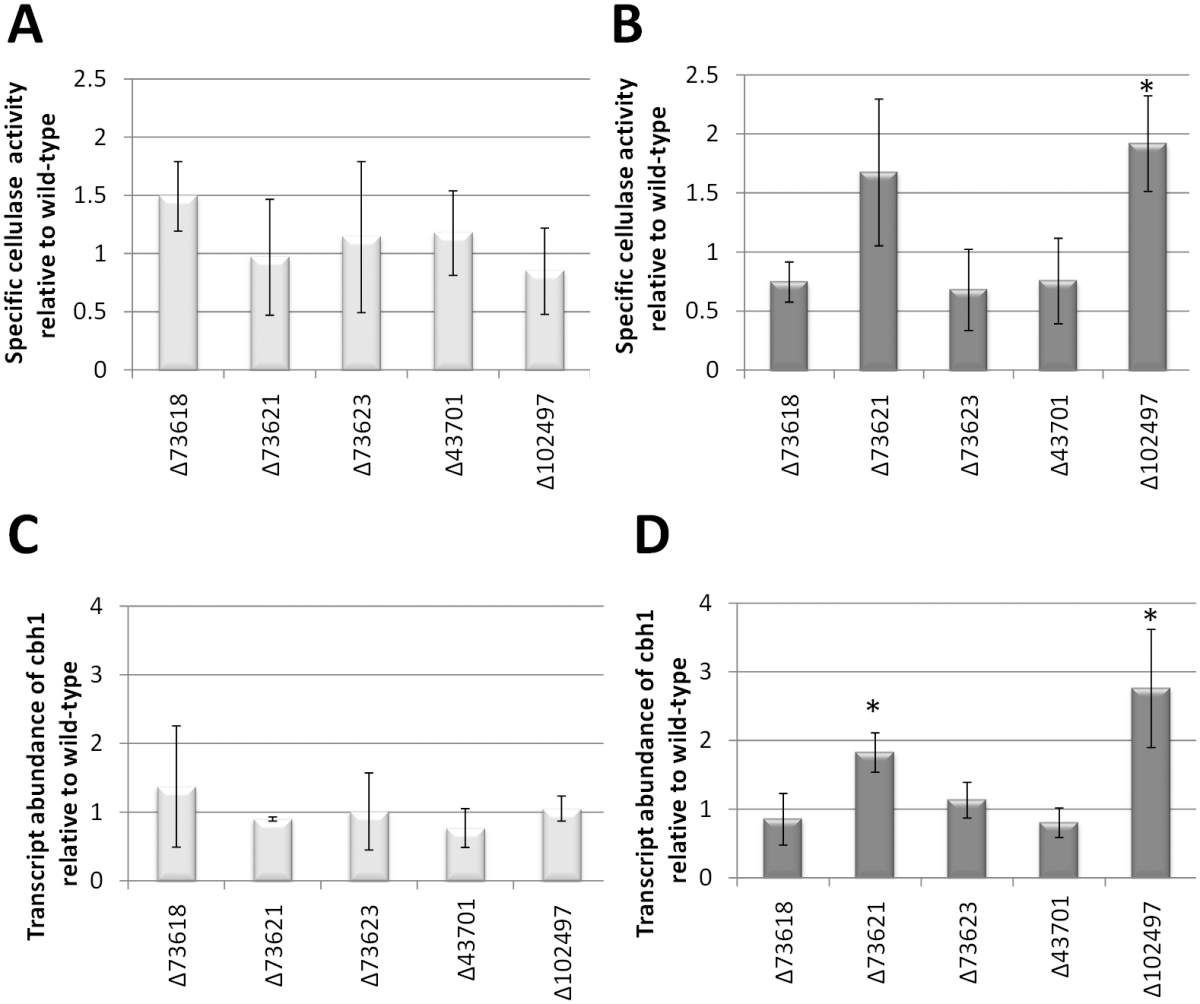
Cellulase regulation by cluster genes. (A, B) Specific cellulase activity in mutants of the cluster genes upon growth in constant light (A, light grey bars) or in constant darkness (B, dark grey bars) for 96 hours on cellulose related to wild-type. (C, D) Transcript levels of *cbh1* in mutants of the cluster genes upon growth in constant light (C, light grey bars) or in constant darkness (D, dark grey bars) for 96 hours on cellulose related to wild-type. Errorbars show standard deviations of at least three biological replicates and two technical replicates. Values with statistically significant difference from wild-type are marked with an asterisk (except for lacking production).

The finding of a secondary metabolite cluster to be regulated by the carbon catabolite repressor CRE1, that is known for its high relevance for cellulase gene expression, suggested a connection of the regulation of this cluster to cellulase gene expression. Therefore we tested all deletion mutants of the cluster genes for their impact on transcript regulation of the major cellulase gene *cbh1* as well as cellulase activity (Fig 6A–6D).

In light, where the transcripts of the cluster genes are only present in very low amounts, and hence likely of low relevance, we did not detect significant changes in *cbh1* transcript levels or specific cellulase activity (Fig 6A and 6C). In darkness however, deletion of the transcription factor gene TR_102497/*ypr2* resulted in increased *cbh1* transcript levels and correspondingly increased specific cellulase activity (Fig 6B and 6D). Deletion of the pks gene TR_73621 resulted in a positive trend for *cbh1* transcript levels and increased specific cellulase activity (Fig 6B and 6D). We conclude that there is indeed a mutual influence of the secondary metabolites produced by this cluster and/or its regulators with enzyme expression.

## Discussion

Polyketides are a diverse group of chemicals being produced as secondary metabolites in many fungi. They include polyphenols, polyenes and macrolides, which can be toxins such as sterigmatocystin or aflatoxin or important pharmaceuticals such as lovastatin [52].

The polyketide synthases found in the SOR cluster belong to the group of non reducing fungal PKS clade III and the reducing clade I of the lovastatin/citrinine type, respectively. Both PKSs are specific to *T*. *reesei* as no orthologues were found in *T*. *atroviride* or *T*. *virens* [42] although there are orthologous genes in *A*. *nidulans* and *N*. *crassa*. The SOR cluster is conserved only in the phylogenetically already relatively distant *Penicillium chrysogenum*, but not in closer related fungi [41, 47].

Previously, regulation of sorbicillin production by the transcription factors YPR1 and YPR2 was shown for growth on glucose [41]. Due to the effect of YPR2 on the sorbicillin derivatives trichodimerol and dihydrotrichotetronin, that we showed here, our data are in line with a biosynthesis of trichodimerol and dihydrotrichotetronin from sorbicillin as intermediate involving the genes of the SOR cluster [41]. It remains to be shown, whether trichodimerol and dihydrotrichotetronin are only produced upon growth on cellulose as in our study or if these compounds were just not detected on glucose. Trichodimerol was isolated from *P*. *chrysogenum* and inhibits production of TNF-alpha by macrophages [53] and exhibits strong cytotoxic activity on three cancer cell lines [54]. Dihydrotrichotetronin was isolated from *Trichoderma longibrachiatum* [55] and is also known as bislongiquinolide [56] or bisorbibutenolide [57]. Also this compound has potential anticancer activity [58, 59].

For trichodimerol and (dihydro)trichodetronin a biosynthetic route was suggested which involves condensation of two sorbyl-cyclohexadienone type 2,4-dimethylhexaketides to form trichodimerol and on the other hand a Diels-Alder reaction for formation of (dihydro)trichotetronine from the same sorbyl-cyclohexadienone and a sorbyl-tetronic acid dimethyl hexaketide [55]. We found that TR_73621 and TR_73623 are essential for both biosynthesis of trichodimerol and dihydrotrichotetronin in light and darkness, while the second PKS TR_73618 is only essential for trichodimerol biosynthesis in darkness. Formation of trichodimerol by the SOR cluster is in agreement with previous studies showing sorbicillin formation [41]. The Diels-Alderase suggested to be required for formation of dihydrotrichotetronine remains to be determined. However, related PKSs have been shown to exert Diels-Alderase activity and also stand-alone Diels Alderases are known [60, 61]. Unfortunately, the structural requirements for enzymatic Diels-Alderase activity are not sufficiently described to conclude a responsible domain and enzymes currently known to show this activity show hardly any sequence similarities [61]. Therefore it can neither be proposed nor excluded that the PKSs of the SOR cluster or TR_73623 could be required for this reaction.

In addition to trichodimerol and dihydrotrichotetronin, an (likely indirect) influence of cluster genes on paracelsin production was observed, which can be assumed to be a side effect due to an imbalance in secondary metabolism and/or precursor availability caused by deletion of the respective genes.

Already with the annotation of the genome of *T*. *reesei* [43], a biased placement of genes involved in secondary metabolism close to CAZyme clusters was noted. At the time it was interpreted as a means to fend off competitors for nutrients. Our study now supports this hypothesis of coordination of substrate degradation and competition and moreover shows that regulation of the SOR cluster is strongly regulated by light. Accordingly, metabolic functions have recently been shown to be considerably influenced by the circadian clock including coordination of anabolic and catabolic functions [62, 63]. Cellulase regulation in *T*. *reesei* is also known to be regulated in dependence of light [7, 10, 12] and transcriptome analysis in light and darkness as well as with photoreceptor mutants also indicated a relevance for energy metabolism [9–11]. Therefore an economic distribution of resources for feeding (enzyme production) and fighting (secondary metabolite production) would appear reasonable.

Nutrient- and light dependent regulation of secondary metabolism has been shown previously and was even dependent on the concentration of a carbon source [46]. Light is also known as one crucial factor in regulation of secondary metabolism in fungi [64]. Hence, the different regulation patterns seen in light and in darkness in our study are not without precedent. However, the mutual regulation of transcript levels in response to the lack of either one of the PKSs or TR_73623 was unexpected and suggests an intracellular sensing mechanism, responding to altered precursor availability or product formation. Despite the numerous reports on regulation of primary and secondary metabolism in light and darkness, a clear explanation as to the biological importance of this regulation, that would also be consistent for different species could not yet been found. Nevertheless, it is tempting to speculate that the different condition in terms of light (including harmful UV light), humidity, oxidative stress etc. during day or night and on versus in the substrate [65] are important for the light dependent gene regulation in fungi.

The function of a switch between primary and secondary metabolism was suggested for *T*. *reesei* XPP1 [45]. XPP1 was initially described as a repressor of xylanase genes, where its deletion caused a roughly 1.5 to 2fold increase in xylanase activity [44]. Interestingly, we see a comparable phenomenon for TR_102497/YPR2, which has a clear influence on secondary metabolism in its gene cluster, but also impacts cellulase gene expression. Even the crucial carbon catabolite repressor CRE1 with its clearly nutrient targeted function impacts regulation of secondary metabolite genes. It will be interesting to learn how widespread the interconnections and regulators of primary and secondary metabolism indeed are. Our findings already indicate an energy driven distribution of resources that is triggered by both regulators of secondary metabolism and primary metabolism.

While CCR is mainly studied with respect to regulation of carbon source degrading enzymes, a function of CCR and/or CRE1-homologues in secondary metabolism was investigated in *Aspergilli*. Although indications for a role of CCR in this process were detected (for example [66]), an involvement of CreA was not unequivocally proven [67, 68]. However, in these studies, the light conditions under which the experiments were performed are not described and a regulatory role of CreA was only analyzed under carbon catabolite repression conditions, but a potential function under derepressed conditions was not considered. In this respect it is also interesting, that a relevance of CRE1 for translation associated functions as found in our study was only detected on glucose so far [69]. Considering the differences in gene regulation and functional distribution of regulated genes [9, 10] under inducing conditions and particularly on cellulose in light and darkness, uncontrolled light conditions may have masked some regulatory effects on cellulose.

## Materials and methods

### Strains and cultivation conditions

QM9414 (ATCC26921), Δ*cre1* [26], QM6a (ATCC13631), QM6aΔ*ku80* and QM6aΔ*mus53* [70] were used. Strains were propagated on malt extract agar (3% w/v; Merck, Darmstadt, Germany).

For transcriptome analysis, Mandels Andreotti (MA) minimal medium [71] supplemented with 0.1% (w/v) peptone (Roth, Karlsruhe, Germany) with 1% (w/v) microcrystalline cellulose (Alfa Aesar, Karlsruhe, Germany) as carbon source. QM9414 (ATCC26921) and Δ*cre1* were grown directly in the MA medium for 72 hours in 200 ml of medium in constant light (1800 lux; Osram L 18W/835 fluorescent bulbs) or constant darkness at 28°C on a rotary shaker (200 rpm). Harvesting was done under red safety light (darkroom lamp, Philips PF712E, red, E27, 15 W) for all dark cultivations in order to avoid interference of light pulses with gene regulation.

The same conditions were applied for cultivation of QM6a, QM6aΔ*ku80* and QM6aΔ*mus53* along with the recombinant strains constructed for this study, except that these strains were grown for 96 hours. Mycelia and supernatants were used for determination of biomass formation, cellulase activity and quantitative reverse transcription PCR (qRT-PCR) analysis. For inoculum production, strains were grown on malt extract agar plates for 10 days in constant darkness to avoid interference of light effects or circadian rhythms. 10^9^ conidia per L were used as inoculum.

### Construction of deletion strains and copy number determination

Yeast recombination cloning was used for vector construction as described [72] using primers for amplification of flanking sequences as provided in this study. Deletions were introduced into QM6aΔku80 (TR_73618, TR_73621, TR_73623 and TR_43701) or QM6a Δmus53 (TR_102497) by protoplasting [73] and absence of open reading frames was confirmed by PCR. Primers used for vector construction and confirmation of deletion are listed in Table A (S3 File). The respective parental strain as well as QM6a were used as controls for every experiment. Determination of copy numbers of integrated deletion cassettes (Table B in S3 File) was performed as described previously [8]. Two deletion strains of the same gene were included in the analysis.

### Nucleic acid isolation and qRT PCR

Strains grown on Mandels Androtti medium with cellulose as carbon source were harvested by filtration and snap frozen in liquid nitrogen. For cultivations in constant darkness, harvesting was done with red safety light. Isolation of total RNA using the RNeasy Plant Mini Kit (QIAGEN, Hilden, Germany) as well as quality control was done as described earlier [17]. Only high quality RNA was used for further analyses. Total RNA (1 μg) was treated with DNase I (Thermo Fisher, Waltham, MA, USA) and reverse transcription was performed using the GoScript Reverse Transcription System (Promega, Madison, USA). qRT-PCR analysis was performed as outlined in (Tisch et al., 2011) with the GoTaq QPCR Master Mix (Promega, Madison, WI, USA) on the CFX96 Real Time cycler (Bio-Rad, Hercules, USA). Three biological replicates and three technical replicates were considered for analysis. Data analysis was done using the software qbase+ (Biogazelle). Primers used are listed in Table A (S3 File).

### Secondary metabolite analysis

For secondary metabolite analysis the same cultures were used as for qRT-PCR, biomass determination and cellulase analysis. Secondary metabolites were determined in the supernatants from these cultures as normalized to the respective biomass formed.

Application of high performance thin layer chromatography (HPTLC) and data visualization was performed as described in [74] except that separation was done with chloroform and 1 mM trifluoroacetic acid in methanol.

Mass spectrometric, quantitative analysis and identification of secreted secondary metabolites was done as described previously [75] with a QTrap 5500 MS/MS system (Applied Biosystems, Foster City, CA) equipped with a TurboIonSpray electrospray ionization (ESI) source and a 1290 series UHPLC system (Agilent Technologies, Waldbronn, Germany). Chromatographic separation was done at 25°C on a Gemini^®^ C18-column, 150×4.6 mm i.d., 5 μm particle size, equipped with a C18 security guard cartridge, 4×3 mm i.d. (all from Phenomenex, Torrance, CA, US). Using this approach, routine detection and quantification of 710 metabolites is performed. Calibration with a serial dilution of a multi analyte stock solution for these metabolites enables reliable identification and quantification of the fungal metabolites present in the sample. Confirmation of positive analyte identification was obtained by the acquisition of two MRMs per analyte, which yielded 4.0 identification points according to commission decision 2002/657/EC. In addition, the LC retention time and the intensity ratio of the two MRM transition agreed with the related values of an authentic standard within 0.1 min and 30% rel., respectively.

### Transcriptome and bioinformatic analysis

We used the gene expression service for custom arrays as provided by Roche-NimbleGen (Madison, USA) for transcriptome analysis of high quality RNA of Δ*cre1* grown in constant light or constant darkness on cellulose. Data are deposited at NCBI Gene Expression Omnibus (GEO accession number GSE99441). For differential gene regulation a threshold of 2fold with a p-value of 0.01, false discovery rate (FDR) corrected, was applied (ANOVA statistics, PARTEK Genomics Suite 6.6; St. Louis, USA).

Functional category analysis was done with the MIPS Functional Catalogue tool in the latest version of May 2014 (http://mips.helmholtz-muenchen.de/funcatDB/; [76]). Analysis of genomic clustering was performed using the open source software REEF [77] and obtained clusters were fused manually if overlapping.

DNA analysis and search for promotor motifs was done with Generunner 3.0 (Version 5.0.79d Beta). The t-test was used to evaluate statistical significance of results.

### Biomass determination of cellulase activity

Determination of biomass formation in liquid culture with cellulose as biomass was done as described previously [7]. Briefly, mycelium was harvested and snap frozen in liquid nitrogen. Then, the material was ground to a fine powder using a Retsch Mill (Retsch MM301, Haan, Germany), resuspended in 0.1 M NaOH, sonicated and after incubation at room temperature and centrifugation, the protein content (reflecting biomass) was determined by the Bradford method (Bio-Rad Protein Assay; Biorad, Hercules, USA). CMCase activity was measured in the culture filtrates using the Azo-CM-Cellulose kit (S-ACMC-L, Megazyme, Wicklow, Ireland).

## Supporting information

S1 File Gene regulation by CRE1 on cellulose in light and darkness. (XLS)

S2 File Genomic clustering of CRE1 regulatory targets. (XLSX)

S3 File Supplementary figure A and supplementary tables A and B. (PDF)
