# Supplementary material for: A CRE1- regulated cluster is responsible for light dependent production of dihydrotrichotetronin in Trichoderma reesei
Source: PLoS One. 2017 Aug 15;12(8):e0182530. doi: 10.1371/journal.pone.0182530 (PMC5557485; doi:10.1371/journal.pone.0182530)
Supplement: S3 File — (PDF) [file pone.0182530.s003.pdf]

A CRE1- regulated cluster is responsible for light dependent production of dihydrotrichotetronin in *Trichoderma reesei*

Alberto Alonso Monroy[1], Eva Stappler[1], Andre Schuster[2], Michael Sulyok[4] and Monika Schmoll[1]

Supplementary material

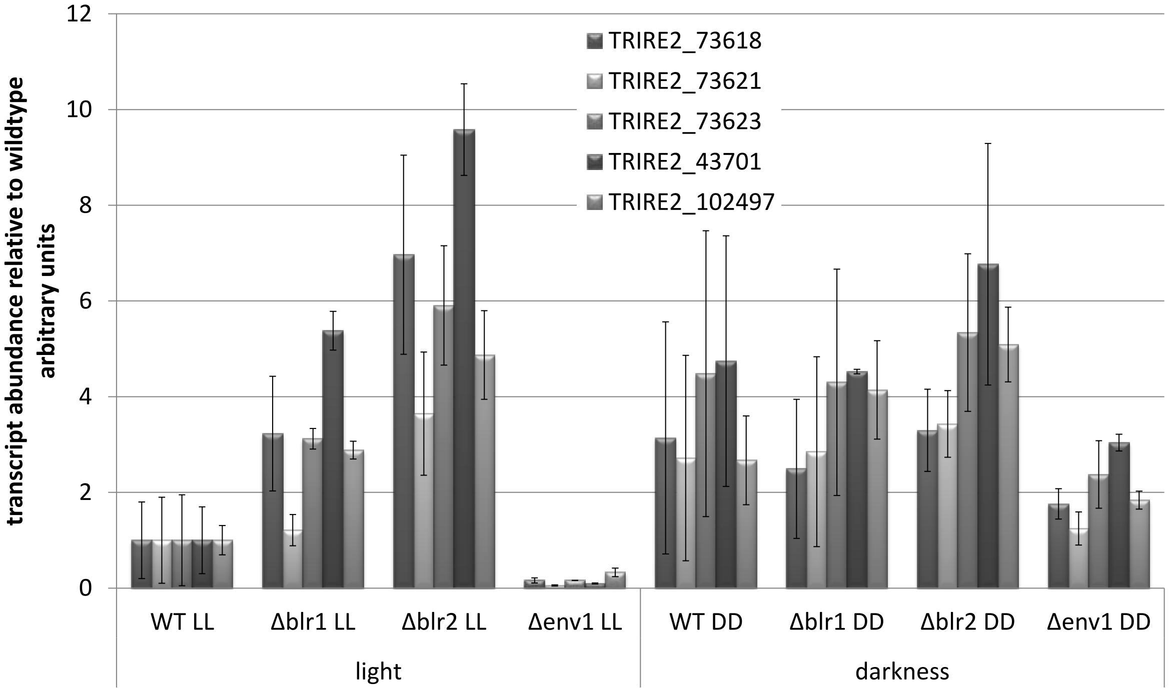

Figure A. Regulation of cluster genes upon growth in light and darkness in wildtype and in deletion mutants of the photoreceptors BLR1, BLR2 and ENV1. Errorbars show standard deviations. Data are taken from (Tisch et al., 2013).

Table A. Oligonucleotides used in this study.

Name	Sequence: 5'-XXXXXX-3'	Purpose	Amplicon size in bp
pdel_43701_5F	GTAACGCCAGGGTTTTCCCAGTCACGACGGTAGAAGGCATCGTAGGTCC	Amplification of 43701 gene 5' flanking region for deletion	1099
pdel_43701_5R	ATCCACTTAACGTTACTGAAATCTCCAACGTGGTTATACTCCGAAGTCC		
pdel_43701_3F	CTCCTTCAATATCATCTTCTGTCTCCGACGCCCTCTCTCATACATCAGC	Amplification of 43701 gene 3' flanking region for deletion	1132
pdel_43701_3R	GCGGATAACAATTTCACACAGGAAACAGCGTGTTCAGCTCTTGAAGTGG		
pdel_53238_5F	GTAACGCCAGGGTTTTCCCAGTCACGACGCATTCTACAGAGGCACTAGG	Amplification of 53238 gene 5' flanking region for deletion	1248
pdel_53238_5R	ATCCACTTAACGTTACTGAAATCTCCAACGAGATGTACGAGTGATGAGC		
pdel_53238_3F	CTCCTTCAATATCATCTTCTGTCTCCGACCTATCCATCTGCCATAGACC	Amplification of 53238 gene 3' flanking region for deletion	1107
pdel_53238_3R	GCGGATAACAATTTCACACAGGAAACAGCCTCCATGCCTAATACCTACC		
pdel_53776_5F	GTAACGCCAGGGTTTTCCCAGTCACGACGACACTCACCTTCTCATCTCC	Amplification of 53776 gene 5' flanking region for deletion	1082
pdel_53776_5R	ATCCACTTAACGTTACTGAAATCTCCAACGACTCCTCCACTTACATTCC		
pdel_53776_3F	CTCCTTCAATATCATCTTCTGTCTCCGACCTATAGTGCTAGACCCCAGC	Amplification of 53776 gene 3' flanking region for deletion	1031
pdel_53776_3R	GCGGATAACAATTTCACACAGGAAACAGCATCTAGCCGTGATACTCTGG		
pdel_73604_5F	GTAACGCCAGGGTTTTCCCAGTCACGACGAGTACCCGACTAATGACTGG	Amplification of 73604 gene 5' flanking region for deletion	1158
pdel_73604_5R	ATCCACTTAACGTTACTGAAATCTCCAACCTCTATTCGTCCCTGCTACC		
pdel_73604_3F	CTCCTTCAATATCATCTTCTGTCTCCGACTCGTGAGAGAGAGAGAGAGG	Amplification of 73604 gene 3' flanking region for deletion	1177
pdel_73604_3R	GCGGATAACAATTTCACACAGGAAACAGCTAGCACCAAGAGTAAGCTCC		
pdel_73618_5F	GTAACGCCAGGGTTTTCCCAGTCACGACGATCTGGCTTGGATACTCACC	Amplification of 73618 gene 5' flanking region for deletion	1216
pdel_73618_5R	ATCCACTTAACGTTACTGAAATCTCCAACGATCAGCAGATCCTGTAAGC		
pdel_73618_3F	CTCCTTCAATATCATCTTCTGTCTCCGACCACACACTCACATCTTCAGC	Amplification of 73618 gene 3' flanking region for deletion	1016
pdel_73618_3R	GCGGATAACAATTTCACACAGGAAACAGCGCTGTACTATCCGTAGAACC		
pdel_73621_5F	GTAACGCCAGGGTTTTCCCAGTCACGACGGTCGAAGAAGTGAGAGATGC	Amplification of 73621 gene 5' flanking region for deletion	1133
pdel_73621_5R	ATCCACTTAACGTTACTGAAATCTCCAACGAGTTGAGATACTCCGATGC		
pdel_73621_3F	CTCCTTCAATATCATCTTCTGTCTCCGACGTAGTCATTTGCTGGTGTGG	Amplification of 73621 gene 3' flanking region for deletion	1081
pdel_73621_3R	GCGGATAACAATTTCACACAGGAAACAGCGTCTTCACTCACCAACATCC		
pdel_73623_5F	GTAACGCCAGGGTTTTCCCAGTCACGACGCTCTTCTCTATCAGCAGTGG	Amplification of 73623 gene 5' flanking region for deletion	1014
pdel_73623_5R	ATCCACTTAACGTTACTGAAATCTCCAACCACACCAGCAAATGACTACC		
pdel_73623_3F	CTCCTTCAATATCATCTTCTGTCTCCGACGCGTAGCATTACTCCATAGC	Amplification of 73623 gene 3' flanking region for deletion	1182
pdel_73623_3R	GCGGATAACAATTTCACACAGGAAACAGCTACGGTCGCTCACTATTAGG		
pdel_73631_5F	GTAACGCCAGGGTTTTCCCAGTCACGACGACCTGAAAGGTAAGGTGACG	Amplification of 73631 gene 5' flanking region for deletion	1123
pdel_73631_5R	ATCCACTTAACGTTACTGAAATCTCCAACAGCTGCTATTGAACCAGAGG		
pdel_73631_3F	CTCCTTCAATATCATCTTCTGTCTCCGACTCCGTTTACACTGAGACAGC	Amplification of 73631 gene 3' flanking region for deletion	1035
pdel_73631_3R	GCGGATAACAATTTCACACAGGAAACAGCCATACGTCCGAAGAGTCACC		
102497_5F	GTAACGCCAGGGTTTTCCCAGTCACGACGGACGCAGTAGATTGTCATGG	Amplification of 102497 gene 5' flanking region for deletion	1088
102497_5R	ATCCACTTAACGTTACTGAAATCTCCAACAGCTACTCATTGGTCAGAGC		
102497_3F	CTCCTTCAATATCATCTTCTGTCTCCGACCTGGTTAAGGTAAGCAGAGC	Amplification of 102497 gene 3' flanking region for deletion	1055
102497_3R	GCGGATAACAATTTCACACAGGAAACAGCGGCCTTACCATTGTGTCTGG		
RT_43701_F	GTCAGCACCATTTGGCTTCGGC	qRT PCR, diagnostic PCR for deletion	264
RT_43701_R	TCCCTGACAACTGCGCCATAGC		
RT_53238_F	AGCGTCATCACCATGTCTCCGC	qRT PCR, diagnostic PCR for deletion	264
RT_53238_R	CGCTTCACTGACGACGAGGGAG		
RT_53776_F	GCGCACTGGAGTATCTGCACGA	qRT PCR, diagnostic PCR for deletion	257
RT_53776_R	CGGTAGCCCTGTCAGCATCTCG		
RT_73604_F	AAGACCTCCCAGCCGACCGATA	qRT PCR, diagnostic PCR for deletion	253
RT_73604_R	AACGTCTCGTACTCGCACCAGC		
RT_73618_F	ATGACGAGGATAGCAAGGCGGC	qRT PCR, diagnostic PCR for deletion	248
RT_73618_R	AATGGACAACTCTGCTCCCGCC		
RT_73621_F	GCAACCTCGTCGATTTGGCTGC	qRT PCR, diagnostic PCR for deletion	218
RT_73621_R	AAGTGTCTCGAGAAGGACGCGC		
RT_73623_F	GACGAGGATGACGTGAAGCGCT	qRT PCR, diagnostic PCR for deletion	266
RT_73623_R	GCCAAGACCAGCGAGTCTTCCA		
RT_73631_F	AACGGCTCCGAAATCACTGCGA	qRT PCR, diagnostic PCR for deletion	232
RT_73631_R	CCCCGGCATCAGATATCGCAGG		
int102497_F1	TGCCTTGGTGGTACCGGGTCTA	qRT PCR, diagnostic PCR for deletion	250
int102497_R1	TCGTCCAAAGCCATGATGCCGT		

Table B. Copy number determination of strains used in this study.

gene ID	strain	copy number
controls	GNA3QL	1
	Δgng1	3
43701	Δ43701 20A	1
	Δ43701 27A	1
53238	Δ53238 18A	1
	Δ53238 40A	1
73604	Δ73604 1A	1
	Δ73604 2B	2
73618	Δ73618 1A	2
	Δ73618 18AA	3
73621	Δ73621 10aa	1
	Δ73621 16aa	3
73623	Δ73623 29A	2
	Δ73623 48B	2
102497	Δ102497 51AA	1
	Δ102497 52AA	1
